# Supplementary material for: Overlooked shelf sediment reductive sinks of dissolved rhenium and uranium in the modern ocean
Source: Nat Commun. 2024 May 10;15:3966. doi: 10.1038/s41467-024-48297-y (PMC11519890; doi:10.1038/s41467-024-48297-y)
Supplement: Supplementary file 1 — Supplementary Information [file 41467_2024_48297_MOESM1_ESM.pdf]

Supplementary Information for

Overlooked shelf sediment reductive sinks of dissolved rhenium and uranium in the modern ocean

Qingquan Hong[1], Yilin Cheng[2,3], Yang Qu[1], Lin Wei[2,3], Yumeng Liu[1], Jianfeng Gao[4], Pinghe Cai[2,3], Tianyu Chen[1*]

1 State Key Laboratory for Mineral Deposits Research, School of Earth Sciences and Engineering and Frontiers Science Center for Critical Earth Material Cycling, Nanjing University, Nanjing 210023, China

2 State Key Laboratory of Marine Environmental Science, Xiamen University, Xiamen 361005, China

3 College of Ocean and Earth Sciences, Xiamen University, Xiamen 361005, China

4 State Key Laboratory of Ore Deposit Geochemistry, Institute of Geochemistry, Chinese Academy of Sciences, Guiyang 550081, China

* Corresponding author: tianyuchen@nju.edu.cn (Tianyu Chen)

This PDF file includes:

Supplementary Texts S1

Supplementary Table S1 to S6

Supplementary Figures S1 to S7

Supplementary References

Text S1 | Enrichment of Re and U in the shelf sediments

We measured Re, U, and Th concentrations of two sediment cores (St. F2-S and B3-W) from the northern and southern subregions after spiking (^{185}Re tracer) and digestion using an HF-HNO_3-HCl mixture. Uranium and Th concentrations were obtained using an iCAP RQ ICP-MS after adding rhodium internal standard, while Re concentrations were measured after purification with AG1-X8 resin. The measured concentrations of the reference standard (MESS-4) fall within 10% of the certified values. Replicate analysis of MESS-4 indicates that the precision is better than 4% (N=4, 1 SD) for all elements. The downcore enrichment of Re and U (Supplementary Fig. S4) is consistent with the downcore reductive removal of porewater Re and U, even though there are elevated concentrations in the upper porewaters (at St. B3-W).

Table S1 | SO_4^{2-}/Cl ratios, NH_4^+, NO_3^-, Fe, Mn, Re, U, and ^{224}Ra ($^{224}Ra_D$) in porewater, as well as total ^{224}Ra ($^{224}Ra_T$) and ^{228}Th activities, and total organic carbon (TOC) in the sediments within the inner shelf of the East China Sea during August 2021.

Depth [cm]	SO_4^{2-}/Cl [mM/M]	NH_4^+ [μmol L^{-1}]	NO_3 [μmol L^{-1}]	Fe [μmol L^{-1}]	Mn [μmol L^{-1}]	Re [pmol L^{-1}]	U [nmol L^{-1}]	$^{224}Ra_D$ [dpm L^{-1}]	$^{224}Ra_T$ [dpm g^{-1}]	^{228}Th [dpm g^{-1}]	$^{224}Ra_T/^{228}Th$	TOC [wt%]
St. F1-S, 27.74°N, 121.06°E; Water depth:13 m; T=28.1 °C; S=32.2; DO penetration: 0.30 cm												
†BW	–	0.6	3.8	‡b.d.	–	39.4	11.0	0.67±0.02	–	–	–	–
0-1	52.79	122.6	15.7	0.29	40.7	49.3	14.0	9.6±1.4	0.41±0.02	0.55±0.01	0.75±0.03	0.83
1-2	52.42	174.9	10.3	0.34	84.2	49.5	16.0	13.1±1.0	0.31±0.01	0.50±0.01	0.63±0.03	0.75
2-3	51.98	171.5	1.7	0.34	100	45.2	17.1	23.5±1.3	0.39±0.01	0.47±0.01	0.83±0.04	0.71
3-4	52.70	191.6	1.2	0.11	113	39.5	16.1	20.1±1.4	0.41±0.02	0.51±0.01	0.81±0.04	0.71
4-5	51.89	236.5	2.1	0.08	158	35.9	16.1	24.5±1.4	0.66±0.02	0.60±0.01	1.11±0.05	0.45
5-6	52.64	189.1	3.0	‡b.d.	149	31.4	18.0	13.1±1.1	0.41±0.02	0.42±0.01	0.98±0.05	0.37
7-8	52.91	182.4	1.0	‡b.d.	160	25.9	17.9	12.9±1.0	0.62±0.02	0.55±0.01	1.14±0.05	0.60
9-10	52.46	177.2	1.0	54.3	239	18.1	4.0	19.0±1.2	0.67±0.02	0.63±0.01	1.07±0.04	0.67
11-12	51.49	185.7	0.9	132	174	12.5	1.4	–	0.71±0.02	0.72±0.02	0.98±0.04	0.48
14-15	50.46	252.1	0.5	139	119	10.1	0.4	11.9±1.0	0.60±0.02	0.68±0.02	0.89±0.04	0.34
19-20	50.78	–	–	77.9	75.0	–	0.4	–	–	–	–	–
24-25	–	–	–	51.6	54.5	–	0.5	–	–	–	–	–
St. F2-S, 27.60°N, 121.28°E; Water depth:28 m; T=25.3 °C; S=33.5; DO penetration: 0.33 cm												
†BW	–	1.1	3.1	‡b.d.	–	40.0	11.5	0.19±0.01	–	–	–	–
0-1	52.47	56.1	0.7	‡b.d.	45.5	39.1	10.4	13.0±1.2	0.90±0.04	1.25±0.03	0.72±0.04	0.57
1-2	52.37	74.6	1.1	‡b.d.	68.4	37.9	10.8	20.6±1.7	0.83±0.03	1.02±0.02	0.81±0.03	0.51
2-3	52.59	117.3	1.3	3.89	134	34.2	11.1	20.0±1.3	0.69±0.02	0.78±0.02	0.89±0.04	0.55
3-4	51.78	191.5	3.0	51.4	142	30.5	7.9	23.8±1.8	0.67±0.02	0.68±0.02	0.99±0.04	0.53
4-5	52.31	170.1	2.8	57.1	135	30.2	5.9	15.8±1.2	0.48±0.02	0.69±0.01	0.70±0.03	0.63
5-6	51.50	236.6	2.6	91.6	121	25.7	5.7	18.2±1.8	0.63±0.02	0.68±0.02	0.92±0.04	0.63
7-8	51.94	265.5	5.8	125	127	25.3	2.1	21.0±1.4	0.66±0.02	0.72±0.02	0.92±0.04	0.56

Table S1 (continued)

Depth [cm]	SO_4^{2-}/Cl [mM/M]	NH_4^+ [µmol L^{-1}]	NO_3 [µmol L^{-1}]	Fe [µmol L^{-1}]	Mn [µmol L^{-1}]	Re [pmol L^{-1}]	U [nmol L^{-1}]	$^{224}Ra_D$ [dpm L^{-1}]	$^{224}Ra_T$ [dpm g^{-1}]	^{228}Th [dpm g^{-1}]	$^{224}Ra_T/^{228}Th$	TOC [wt%]
9-10	51.65	239.4	1.3	111	135	19.0	1.0	22.4±1.6	0.74±0.03	0.74±0.02	1.00±0.04	0.50
11-12	51.48	267.4	6.3	160	141	15.4	0.7	–	0.67±0.02	0.59±0.01	1.14±0.05	0.38
14-15	51.16	320.1	7.4	148	113	12.4	0.5	19.1±1.6	0.53±0.02	0.58±0.01	0.92±0.04	0.49
19-20	50.83	–	–	84.4	78.9	–	0.4	–	–	–	–	–
24-25	51.00	–	–	38.4	87.6	–	0.5	–	–	–	–	–
St. F3-S, 27.44°N, 121.56°E; Water depth:48 m; T=22.7 °C; S=33.8; DO penetration: 0.15 cm												
†BW	–	1.4	0.3	‡b.d.	–	39.4	11.7	0.06±0.00	–	–	–	–
0-1	50.57	70.0	14.9	0.12	60.2	46.6	13.2	5.4±0.5	0.48±0.02	0.66±0.01	0.72±0.03	0.56
1-2	49.95	89.9	12.7	2.12	129	43.9	11.2	8.3±0.7	0.52±0.02	0.63±0.01	0.83±0.03	0.68
2-3	51.85	127.6	13.0	16.6	132	40.1	11.3	8.0±0.7	0.50±0.02	0.57±0.01	0.88±0.04	0.70
3-4	49.95	117.2	13.8	32.9	135	39.7	5.2	7.1±0.8	0.56±0.02	0.51±0.01	1.08±0.05	0.65
4-5	50.23	116.8	11.3	21.6	155	35.7	9.6	5.4±0.6	0.72±0.02	0.75±0.02	0.96±0.04	0.67
5-6	50.00	139.1	11.6	42.8	147	28.6	5.2	6.6±0.9	0.69±0.03	0.70±0.02	0.97±0.04	0.70
7-8	49.18	143.9	9.5	80.0	92.1	22.0	1.0	13.2±0.9	0.69±0.02	0.71±0.02	0.97±0.04	0.65
9-10	49.07	170.9	10.2	72.6	106	15.8	1.4	12.9±0.8	0.50±0.02	0.59±0.01	0.85±0.04	0.62
11-12	49.04	118.4	8.5	66.4	95.2	15.7	0.6	–	0.66±0.02	0.63±0.01	1.05±0.04	0.65
14-15	49.26	193.1	7.6	54.9	87.2	9.8	0.6	12.8±0.9	0.56±0.02	0.58±0.01	0.97±0.04	0.67
19-20	49.51	–	–	39.2	85.4	5.2	2.8	–	–	–	–	–
24-25	49.32	–	–	30.1	67.6	2.8	0.8	–	–	–	–	–
St. F4-S, 27.31°N, 121.79°E; Water depth:82 m; T=21.8 °C; S=33.9; DO penetration: 0.48 cm												
†BW	–	1.1	1.2	‡b.d.	–	38.5	12.2	0.04±0.00	–	–	–	–
0-1	52.15	49.1	2.3	‡b.d.	18.9	37.8	11.3	7.6±0.8	0.55±0.02	0.63±0.01	0.87±0.03	0.73
1-2	52.26	67.5	1.6	‡b.d.	136	35.0	12.3	5.1±0.8	0.43±0.01	0.49±0.01	0.88±0.03	0.68

Table S1 (continued)

Depth [cm]	SO_4^{2-}/Cl [mM/M]	NH_4^+ [µmol L^{-1}]	NO_3 [µmol L^{-1}]	Fe [µmol L^{-1}]	Mn [µmol L^{-1}]	Re [pmol L^{-1}]	U [nmol L^{-1}]	$^{224}Ra_D$ [dpm L^{-1}]	$^{224}Ra_T$ [dpm g^{-1}]	^{228}Th [dpm g^{-1}]	$^{224}Ra_T/^{228}Th$	TOC [wt%]
2-3	52.34	78.8	1.3	‡b.d.	163	32.3	12.5	16.2±1.2	0.44±0.02	0.54±0.01	0.82±0.03	0.76
3-4	52.22	96.8	0.7	1.27	139	28.4	9.4	17.7±1.5	0.59±0.02	0.59±0.01	1.01±0.04	0.73
4-5	52.70	111.8	0.4	8.42	89.7	29.1	5.8	16.6±1.3	0.54±0.02	0.54±0.01	1.00±0.04	0.63
5-6	52.12	106.4	0.6	50.6	71.2	23.1	3.3	16.0±1.7	0.60±0.02	0.62±0.01	0.97±0.04	0.62
7-8	51.99	103.5	0.2	99.7	63.1	16.5	1.6	15.3±1.2	0.52±0.02	0.52±0.01	1.01±0.04	0.68
9-10	51.83	119.5	0.2	74.1	49.8	11.1	1.2	15.7±1.2	0.41±0.02	0.45±0.01	0.92±0.04	0.67
11-12	51.68	98.0	0.8	76.0	47.0	6.5	1.2	–	0.55±0.02	0.59±0.01	0.94±0.04	0.75
14-15	51.11	145.3	0.6	77.2	42.5	3.6	0.8	12.7±1.2	0.43±0.02	0.52±0.01	0.83±0.04	0.72
19-20	50.64	–	–	83.0	40.3	2.8	0.8	–	–	–	–	–
24-25	50.53	–	–	71.7	42.0	3.0	1.0	–	–	–	–	–

* BW: Bottom Water; $^{224}Ra_T$ and ^{228}Th denote measurements in the suspended particles.

–: Not available

‡ b.d.: Concentrations indiscernible from the procedure blank of dissolved Fe (66±3 nmol L^{-1}).

Table S2 | Porewater SO_4^{2-}/Cl ratio Mn, Re, and U in the shallow sediments within the shelf of the East China Sea during August 2017 and December 2018.

Depth [cm]	SO_4^{2-}/Cl ratio [mM M^{-1}]	Mn [μmol L^{-1}]	Re [pmol L^{-1}]	U [nmol L^{-1}]
St. Y3-S, 31.06°N, 122.37°E; Water depth: 14 m; T=25.1 °C; S=25.9; DO penetration: 0.22 cm				
†BW	–	‡b.d.	–	–
0-1	–	7.1	27.3	–
1-2	–	47.6	16.6	–
2-3	–	83.7	11.5	–
3-4	–	171	13.5	–
4-5	–	176	16.2	–
5-6	–	190	16.5	–
7-8	–	135	14.4	–
9-10	–	247	13.8	–
11-12	–	220	14.5	–
14-15	–	232	13.7	–
St. Y4-S, 30.86°N, 122.66°E; Water depth: 17 m; T=23.9 °C; S=29.9; DO penetration: 0.29 cm				
†BW	–	–	–	–
0-1	51.20	22.3	41.1	5.9
1-2	51.16	19.6	41.3	4.6
2-3	51.41	18.6	42.8	4.5
3-4	51.38	23.3	37.2	4.6
4-5	51.26	20.9	39.9	3.2
5-6	51.29	29.8	36.7	2.4
7-8	51.27	38.6	32.6	1.4
9-10	51.22	53.6	28.7	2.6
11-12	50.60	47.0	20.1	1.8
14-15	50.55	39.3	18.4	1.9
St. Y5-S, 30.94°N, 122.96°E; Water depth: 47 m; T=22.0 °C; S=34.6; DO penetration: 0.19 cm				
†BW	–	–	–	–
0-1	51.60	23.8	–	10.3
1-2	51.00	9.9	–	9.1
2-3	51.45	9.4	–	9.3
3-4	51.24	7.5	–	8.9
4-5	51.16	10.2	–	5.7
5-6	51.42	12.0	–	4.3
7-8	50.64	23.0	–	1.9
9-10	50.86	27.3	–	2.4
11-12	50.60	20.8	–	1.3
St. Y7-S, 30.79°N, 123.19°E; Water depth: 58 m; T=22.0 °C; S=35.0; DO penetration: 0.31 cm				
†BW	–	–	–	–
0-1	51.52	6.8	50.5	11.9

Table S2 (continued)

Depth [cm]	SO_4^{2-}/Cl ratio [mM M^{-1}]	Mn [μmol L^{-1}]	Re [pmol L^{-1}]	U [nmol L^{-1}]
1-2	51.31	5.5	51.6	14.3
2-3	51.58	11.5	54.4	16.2
3-4	52.46	10.0	77.9	18.6
4-5	51.47	14.1	–	27.3
5-6	51.18	11.8	114.6	27.1
7-8	51.57	10.0	56.4	9.8
9-10	51.41	12.8	–	14.9
11-12	50.62	7.8	28.6	7.2
14-15	51.28	8.9	–	10.1
St. E14-S, 30.75°N, 122.76°E; Water depth: 27 m; T=21.1 °C; S=33.8; DO penetration: 0.30 cm				
†BW	–	–	–	–
0-1	52.15	114	43.7	6.9
1-2	53.15	163	41.0	5.9
2-3	51.52	176	43.3	6.3
3-4	51.42	172	45.3	6.4
4-5	51.65	203	39.4	6.4
5-6	51.53	190	43.6	5.4
7-8	51.49	223	33.6	3.1
9-10	51.50	258	31.0	5.5
11-12	51.21	307	26.3	4.5
14-15	51.11	323	21.5	4.2
St. E13-S, 30.08°N, 122.73°E; Water depth: 40 m; T=21.0 °C; S=34.0; DO penetration: 0.12 cm				
†BW	–	–	–	–
0-1	52.26	168	47.9	5.5
1-2	50.13	92.0	37.3	3.3
2-3	50.63	73.1	32.0	2.4
3-4	50.63	79.9	32.7	3.8
4-5	50.47	106	34.2	2.5
5-6	50.61	114	30.0	2.8
7-8	50.58	71.5	29.4	1.3
9-10	50.71	50.2	23.0	0.9
11-12	51.46	33.8	22.2	1.9
14-15	50.28	31.1	21.2	3.0
St. E10-S, 29.66°N, 122.83°E; Water depth:34 m; T=23.7 °C; S=34.6; DO penetration: 0.43 cm				
†BW	–	–	–	–
0-1	50.51	43.7	–	7.4
1-2	51.28	107	–	5.9
2-3	51.49	77.6	–	3.7
3-4	51.66	48.6	–	2.7

Table S2 (continued)

Depth [cm]	SO_4^{2-}/Cl ratio [mM M^{-1}]	Mn [μmol L^{-1}]	Re [pmol L^{-1}]	U [nmol L^{-1}]
4-5	51.68	39.0	–	3.1
5-6	51.06	30.2	–	1.8
7-8	52.37	38.5	–	1.5
9-10	51.56	29.9	–	1.6
11-12	51.42	27.3	–	1.9
14-15	50.75	25.9	–	2.4
St. E4-S, 28.74°N, 122.00°E; Water depth:17 m; T=26.3 °C; S=33.3; DO penetration: 0.30 cm				
†BW	–	–	–	–
0-1	52.01	74.1	48.7	11.2
1-2	51.75	56.7	43.7	11.2
2-3	52.02	34.9	38.4	11.0
3-4	51.75	58.9	36.1	12.6
4-5	51.77	86.0	38.4	13.2
5-6	51.67	79.7	38.7	13.3
7-8	51.18	46.9	31.6	9.9
9-10	51.41	35.7	–	3.1
11-12	51.55	42.9	28.3	2.7
14-15	50.66	219	25.7	0.4
St. E3-S, 28.00°N, 121.78°E; Water depth:34 m; T=23.7 °C; S=34.6; DO penetration: 0.24 cm				
†BW	–	–	–	–
0-1	–	22.9	39.2	14.9
1-2	–	116	39.0	11.9
2-3	–	92.3	36.8	11.0
3-4	–	117	35.5	8.9
4-5	–	96.8	26.1	7.7
5-6	–	62.1	29.7	7.1
7-8	–	51.3	22.1	7.4
9-10	–	46.2	22.9	6.9
11-12	–	81.1	15.6	2.1
14-15	–	57.7	12.6	2.1
St. A3-W, 33.12°N, 122.41°E; Water depth:27 m; T=16.6 °C; S=32.2; DO penetration: 0.51 cm				
†BW	–	–	41.6	10.2
0-1	51.21	3.5	43.0	12.3
1-2	50.90	30.0	–	10.3
2-3	51.05	54.2	36.9	3.7
3-4	50.85	63.0	25.8	1.8
4-5	50.44	58.6	–	10.1
5-6	50.40	56.8	24.3	6.5
7-8	–	72.2	–	8.0

Table S2 (continued)

Depth [cm]	SO_4^{2-}/Cl ratio [mM M^{-1}]	Mn [μmol L^{-1}]	Re [pmol L^{-1}]	U [nmol L^{-1}]
9-10	–	64.6	–	5.6
11-12	49.88	33.7	14.2	9.8
14-15	–	19.0	–	8.0
St. B3-W, 32.52°N, 122.40°E; Water depth:28 m; T=17.1 °C; S=31.7; DO penetration: 0.45 cm				
†BW	–	–	40.6	9.7
0-1	51.12	0.0	37.0	7.2
1-2	51.07	7.5	36.8	7.3
2-3	50.91	10.4	47.8	9.1
3-4	50.90	9.6	84.2	17.1
4-5	50.80	10.2	57.4	8.5
5-6	50.95	10.2	44.4	8.3
7-8	50.81	13.4	40.6	3.8
9-10	50.82	22.1	39.9	5.6
11-12	50.75	25.4	34.8	2.4
14-15	50.46	30.5	16.0	1.2
17-18	50.23	29.1	–	2.6
St. D2-W, 31.37°N, 122.56°E; Water depth:17 m; T=19.5 °C; S=32.0; DO penetration: 0.15 cm				
†BW	–	–	41.4	9.9
0-1	50.51	0.0	44.4	5.5
1-2	50.23	8.5	44.3	5.4
2-3	49.45	14.4	44.1	4.1
3-4	49.44	15.2	41.3	3.4
4-5	49.01	20.6	37.1	1.2
5-6	48.49	13.0	34.8	1.2
7-8	46.73	9.8	32.9	0.9
9-10	45.73	8.2	29.3	1.5
11-12	44.09	5.6	24.5	2.5
14-15	40.20	5.6	23.0	0.6
St. S1-W, 31.01°N, 122.82°E; Water depth:29 m; T=19.5 °C; S=31.6; DO penetration: 0.18 cm				
†BW	–	–	40.4	9.6
0-1	51.14	78.5	39.2	7.7
1-2	50.56	80.3	37.3	5.3
2-3	50.40	80.0	29.7	3.9
3-4	50.16	66.5	25.8	1.8
4-5	49.97	58.7	20.5	0.8
5-6	49.78	49.2	20.3	0.7
6-7	50.68	51.5	24.2	1.1
7-8	49.76	47.0	20.6	0.9
8-9	49.70	–	15.3	–

Table S2 (continued)

Depth [cm]	SO_4^{2-}/Cl ratio [mM M^{-1}]	Mn [μmol L^{-1}]	Re [pmol L^{-1}]	U [nmol L^{-1}]
9-10	49.44	45.2	13.6	3.9
11-12	49.39	46.6	13.9	0.7
13-14	49.36	34.6	–	1.7
14-15	–	34.0	–	–
17-18	49.20	–	8.9	0.9
19-20	48.73	38.2	4.7	0.7
21-22	48.86	32.1	5.9	1.2
23-24	48.56	31.2	–	2.0
St. E3-W, 30.54°N, 122.81°E; Water depth:37 m; T=20.9 °C; S=33.6; DO penetration: 0.42 cm				
†BW	–	–	42.9	9.6
0-1	50.86	11.1	48.7	9.2
1-2	50.67	13.5	42.3	8.1
2-3	50.46	72.4	38.2	6.0
3-4	50.22	101	29.2	3.8
4-5	50.03	113	26.7	3.7
5-6	49.67	123	19.9	2.9
7-8	49.57	106	18.1	1.7
9-10	49.60	91.1	19.1	1.1
11-12	49.90	62.6	19.6	0.9
13-14	–	47.0	–	1.1
14-15	50.18	–	18.5	–
17-18	–	34.8	10.5	0.4
19-20	–	27.1	4.6	1.2
St. E1-W, 30.70°N, 122.53°E; Water depth:14 m; T=17.5 °C; S=22.3; DO penetration: 0.69 cm				
†BW	–	–	48.5	8.7
0-1	50.62	4.5	44.8	5.2
1-2	50.50	7.3	45.1	2.1
2-3	50.39	–	40.3	1.2
3-4	50.31	14.6	36.2	0.8
4-5	48.99	15.2	31.8	1.0
5-6	49.14	13.4	26.3	0.3
7-8	47.60	13.0	21.5	1.6
9-10	47.71	13.1	16.3	0.6
11-12	46.71	–	10.2	0.8
13-14	–	13.0	6.6	1.1
14-15	46.75	–	–	–
15-16	46.51	–	10.7	1.4
17-18	–	–	2.7	0.4

*Dissolved NH_4^+, NO_3^-, Fe, ^{224}Ra ($^{224}Ra_D$) in porewater, and total ^{224}Ra ($^{224}Ra_T$) and ^{228}Th activities during these two surveys have been published elsewhere[1, 2]. Here, we use these measurements to

decipher the sediment redox conditions.

† BW: Bottom Water.

‡ b.d.: Measurements indiscernible from the mean procedure blank of dissolved Fe, which was determined to be 66±3 nmol L^{-1}.

–: Not available.

Table S3 | Comparison of key environmental variables between globally representative oceanic shelves and the regions chosen for extrapolation. The values are displayed as a range with the average in parentheses.

Variable[a,b]	Unit	East China Sea	Southern Massachusetts	Mid-Atlantic Bight	North American and East Siberian Arctic margin	Hudson Bay	Global oceanic shelves
		This study and refs. [3, 4, 5]	refs. [6, 7, 8]	refs. [6, 9]	refs. [9, 10, 11, 12, 13]	refs.[14, 15]	refs. [3, 9, 16, 17]
Water depth	m	13–82	5	75	12–186	119–200	0–200 (65)
Sedimentation rate	mg cm^{-2} yr^{-1}	200–5000 (380)	100–270	33	90–750[c]	30–130	496
Org. C rain rate	mmol C m^{-2} d^{-1}	4.4–42.7 (15.2)	14.5–43.8	4.4	4.6–40[d]	2.3–4.0	13.4
Org. C decomposition rate	mmol C m^{-2} d^{-1}	3.6–17.6 (9.4)	10.9–24.1	3.3	2.0–44.4	0.6–1.2	9.4

a: these variables on sedimentary and diagenetic environments might modulate the sedimentary reductive removal of Re and U and are thus chosen;

b: the values are displayed as the range and/or average as the data were initially reported in different forms;

c: calculated with the ^{210}Pb-derived sedimentation rate (cm yr^{-1}) and a sediment density of 2.5 g cm^{-3} (refs.[10, 13]);

d: calculated with the sedimentation rate and the TOC contents in the surface sediments (refs.[10, 13]).

Table S4 | Sedimentary reductive removal of Re and U between globally representative sinks and our study (Positive and negative values denote fluxes into and out of the sediment). The uncertainties in this study were propagated from the errors associated with the ^{224}Ra fluxes and the concentration gradients of dissolved ^{224}Ra, Re, and U, while no uncertainties were reported in the literature.

	Station	Bottom Depth [m]	Re [pmol m^{-2} d^{-1}]	U [nmol m^{-2} d^{-1}]	Reference
[a]East China Sea	Y3-S	14	720±210	–[c]	This study
	Y4-S	17	110±23	11±2.4	
	Y5-S	47	–[c]	49±12	
	Y7-S	58	–[d]	–[d]	
	E14-S	27	20±5.1	1.5±0.6	
	E13-S	40	82±19	6.3±1.8	
	E10-S	52	–[c]	34±14	
	E4-S	17	26±16	–[d]	
	E3-S	34	53±22	13±5.5	
	F1-S	13	65±23	–[d]	
	F2-S	28	120±22	22±4.6	
	F3-S	48	320±66	44±13	
	F4-S	82	320±61	56±14	
	A3-W	27	1100±260	–[d]	
	B3-W	28	–[d]	–[d]	
	D2-W	17	510±64	91±19	
	S1-W	29	900±180	110±20	
	E3-W	37	380±140	36±13	
	E1-W	14	4600±950	830±320	
[a]Southern Massachusetts	Hingham Bay	5	39	31	[8]
		5	51	15	
		5	69	6.7	
	Buzzards Bay	15	190	19	[7]
		15	159	50	
		15	69	37	
		15	149	47	
[a]Mid-Atlantic Bight	OC426	75	8.0	–	[6]
[b]North American Arctic margin	SLIP1	80	605	106	[10]
	SLIP3	73	218	43	

	SLIP4	73	352	48	
	UNT3	50	131	52	
	UNT5	51	131	51	
	UNT7	58	115	49	
	BC3	186	65	29	
	QM1	113	88	35	
	FS1	141	18	8.0	
[b]Hudson Bay	4	153	56	6.9	[14]
	6	119	21	8.1	
	8	150	51	15	
	10	200	24	4.6	
[b]East Siberian Arctic Shelves	LV 77-12	37	–	10	[13]
	LV 77-14	40	–	30	
	LV 77-21	43	–	30	
	LV 77-33	46	–	10	
	LV 77-40	12	–	48	
	LV 77-43	21	–	32	
	LV 77-1	44	–	23	
	LV 77-5	51	–	30	
	14R07	73	–	12	
Compiled flux	**Shelf sediment**		112 [120, 299][e]	30 [25, 41][e]	This study
	[b]Oxic		0.24	22.9	[18, 19, 20]
	[b]Suboxic		62	1.9	
	[b]Anoxic		197	25.2	

a: The reductive removal rates were estimated from measurement of porewater: sediment incubation and model fit (literature data) and the $^{224}Ra/^{228}Th$ disequilibrium approach (the East China Sea);
b: Accumulation rates of authigenic Re and U;
c: No data due to limited sample size;
d: The flux cannot be calculated due to the complicated removal-release in the upper sediments indicated by distinct zig-zag patterns within the upper few cm;
e: The compiled data reported as median [lower bound, upper bound of 95% confidence interval].

Table S5 | Measured element concentrations±standard deviation of Certified Reference Material (CRM: SLRS-6 and MESS-4) and dilute seawater standard (NASS-7) spiked with Fe and Mn.

		Fe [ppb]	Mn [ppb]	Re [ppt]	U [ppb]	Reference
SLRS-6 (River water)	Certified	84.5±3.6	2.12±0.10	13.5	0.0699±0.0034	[21, 22, 23]
	Measured	83.5±4.0 (n=19)	2.14±0.10 (n=19)	14.1±0.22 (n=6)	0.0679±0.0027 (n=19)	This study
Spiked NASS-7	Certified	4.90[a]	4.90[a]	6.4	2.81	[24]
	Measured	4.58±0.14 (n=23)	4.52±0.06 (n=23)	6.39±0.13 (n=6)	2.87±0.06 (n=23)	This study
MESS-4 (Marine sediment)[b]	Certified	–	–	2.68–4	3.4±0.4	[25]
	Measured	–	–	3.01±0.12 (n=4)	3.48±0.08 (n=4)	This study

a: predicted values for the spiked seawater standard;

b: the units for MESS-4 are ppb for Re, and ppm for U.

Table S6 | Concentration gradients of dissolved ^{224}Ra, Re, and U used for the flux estimation. The gradients of ^{224}Ra for the cruises in 2017 and 2018 are adopted from refs. [1, 2].

Station	$\partial C^{Ra}/\partial z$ [a] [dpm L^{-1} cm^{-1}]	$\partial C^{Re}/\partial z$ [a] [pM cm^{-1}]	$\partial C^{U}/\partial z$ [a] [nM cm^{-1}]
Summer 2017			
Y3-S	0.014±0.002	-7.88±1.60	–[b]
Y4-S	0.029±0.002	-1.86±0.18	-0.62±0.07
Y5-S	0.017±0.001	–[b]	-1.55±0.27
Y7-S	0.015±0.001	–[c]	–[c]
E14-S	0.040±0.003	-1.71±0.22	-0.43±0.14
E13-S	0.030±0.002	-1.57±0.28	-0.41±0.10
E10-S	0.011±0.001	–[b]	-1.62±0.16
E4-S	0.029±0.002	-1.44±0.21	–[c]
E3-S	0.015±0.002	-2.04±0.19	-1.73±0.18
Summer 2021			
F1-S	0.048±0.003	-3.58±0.14	–[c]
F2-S	0.041±0.003	-1.97±0.12	-1.24±0.14
F3-S	0.017±0.001	-3.55±0.20	-1.65±0.35
F4-S	0.015±0.002	-2.93±0.12	-1.73±0.30
Winter 2018			
A3-W	0.018±0.001	-2.49±0.57	–[c]
B3-W	0.010±0.001	–[c]	–[c]
D2-W	0.029±0.002	-1.74±0.12	-1.06±0.20
S1-W	0.018±0.002	-4.24±0.46	-1.74±0.13
E3-W	0.022±0.002	-5.71±0.32	-1.82±0.18
E1-W	0.006±0.001	-3.17±0.15	-1.96±0.65

a: negative values denote decreasing concentrations downcore (i.e., sedimentary reductive removal);
b: not available due to limited sample size;
c: the distinct zig-zag patterns within the upper few cm, indicating complicated removal-release in the upper sediments, hinder reliable calculation of their gradients and reductive removal fluxes.

Fig. S1 | Sampling locations in the East China Sea. Red and blue symbols denote summer and winter, respectively. This figure was produced using Ocean Data View[26].

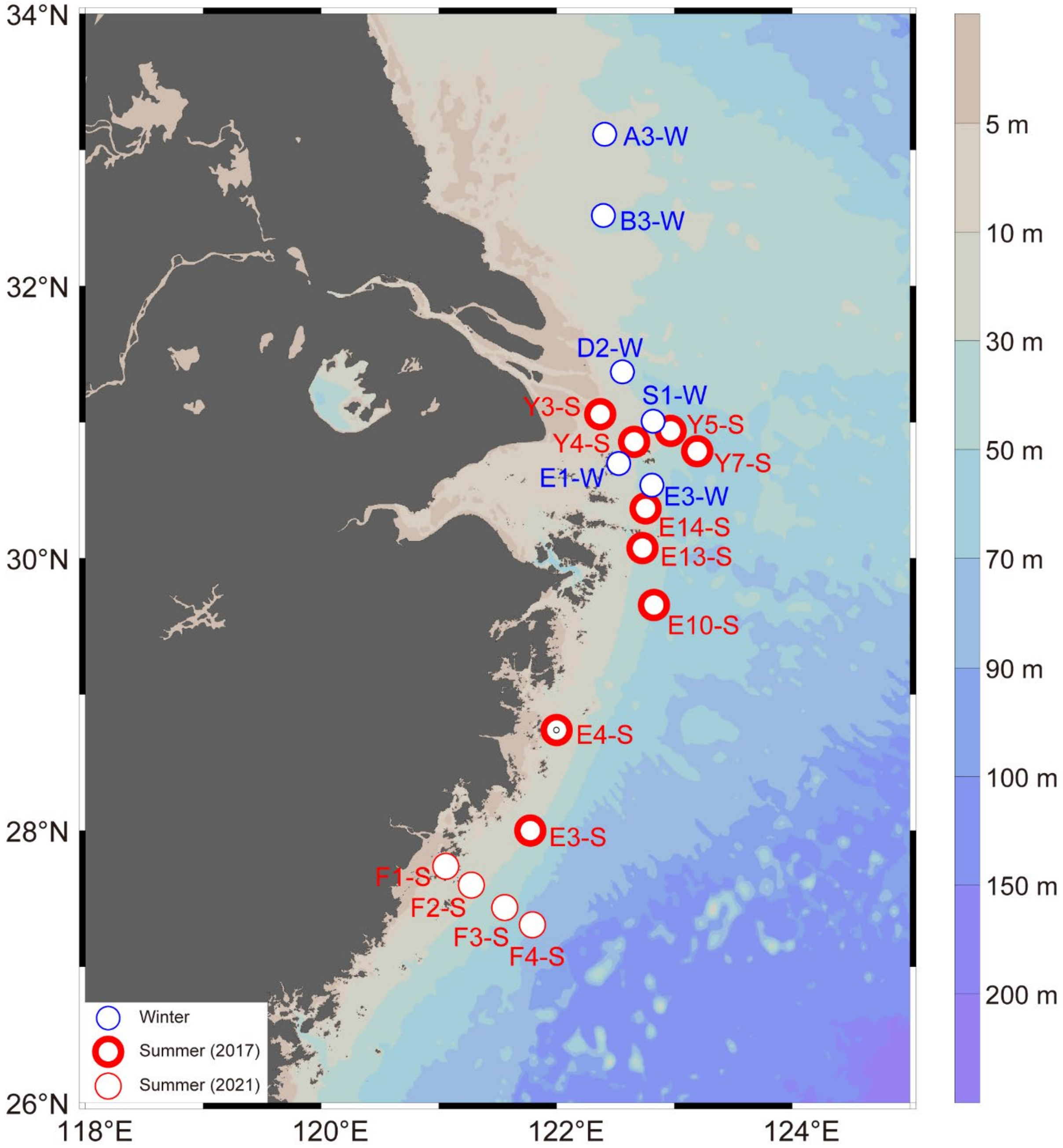

Fig. S2 | Porewater geochemistry in the sediments of the East China Sea. Groups **a-e** SO_4^{2-}/Cl ratio, NO_3^-, NH_4^+, Fe, and Mn versus depth. The horizontal dash line denotes the sediment–water interface.

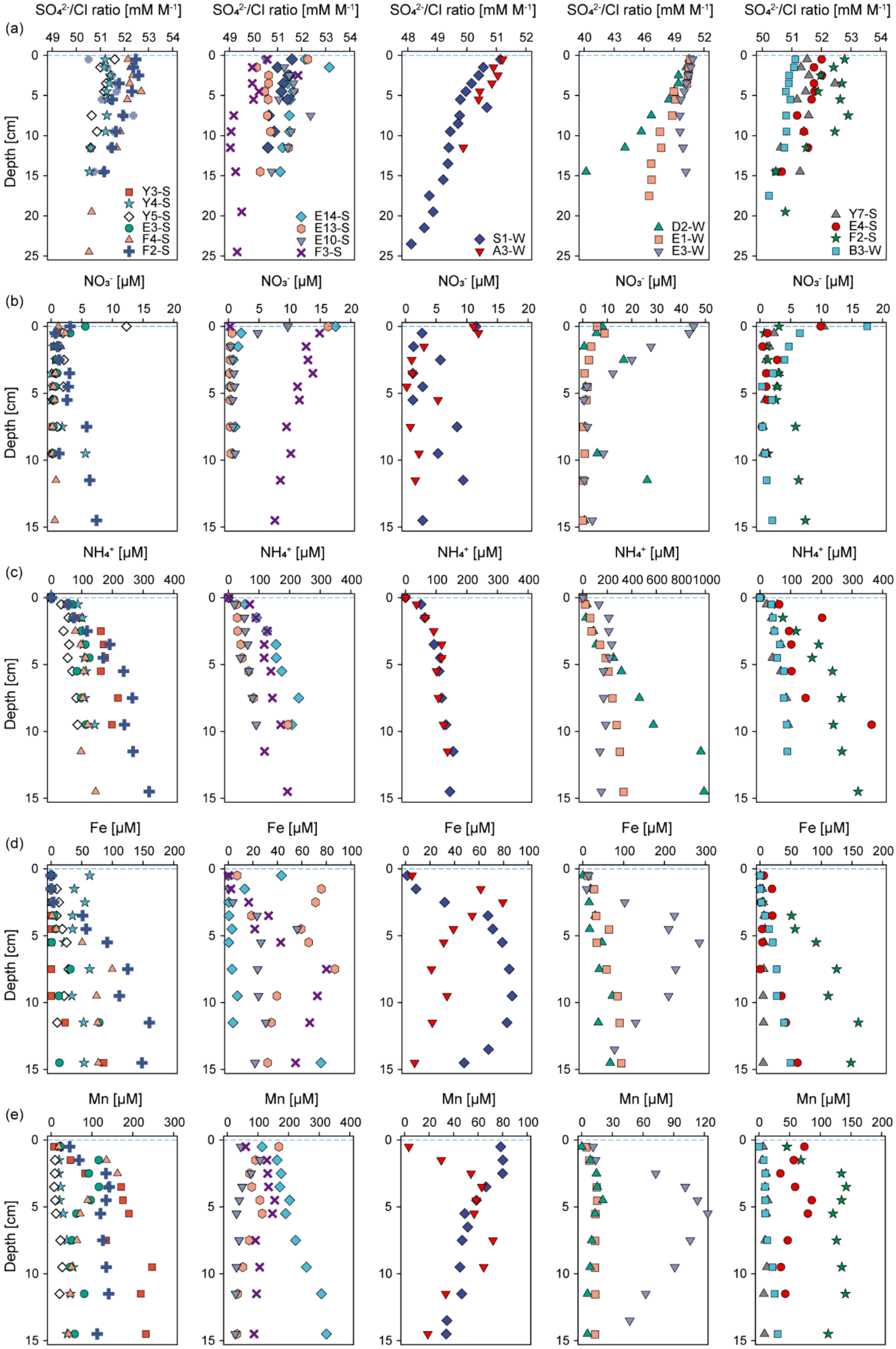

Fig. S3 | Concentrations of Re and U in the water column of the East China Sea. Squares denote the bottom water collected during the winter of 2018; circles and diamonds denote the vertical profiles collected in the summer of 2019 and 2021. The gray bar represents the average±1 standard deviation (N=23) of the samples. The elevated (Re) and lower (U) concentrations (circled) are at St. E1-W, which is located near a refuge harbor.

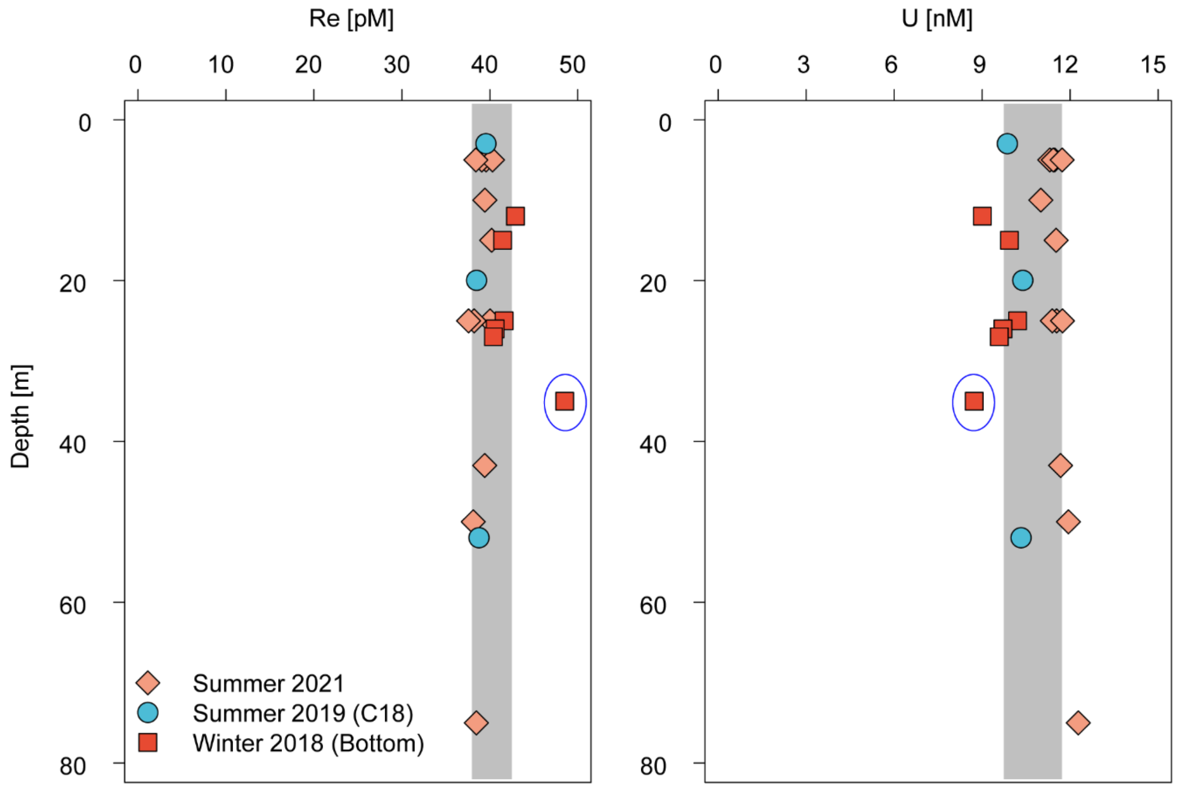

Fig. S4 | Solid phase profiles of Re/Th and U/Th ratios from St. F2-S (southern site) and St. S1-W (middle site) of the East China Sea shelf. The profiles show the enrichment features of U and Re versus depth.

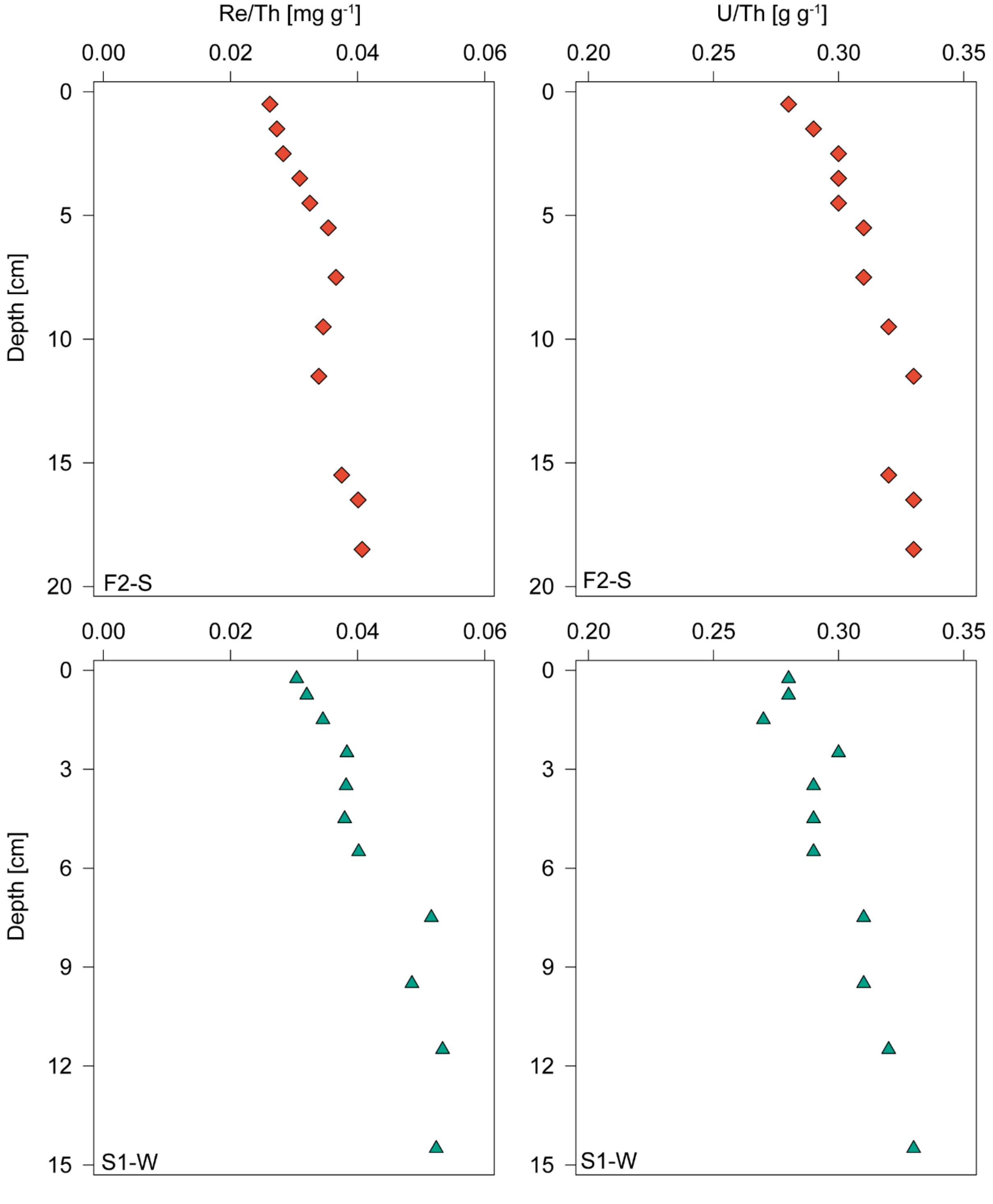

Fig. S5 | Schematic of Re and U input and subsequent reductive removal in the sediments (not scaled). Scenario 1: reductive removal exceeds the release of dissolved Re and U from particles at the sediment–water interface; Scenario 2: release from particles far exceeds reductive removal at the sediment–water interface, but reductive removal prevails and uptakes the released Re and U below; Scenario 3: Re and U release from particles exceeds reductive removal and accumulate in porewater over a depth interval.

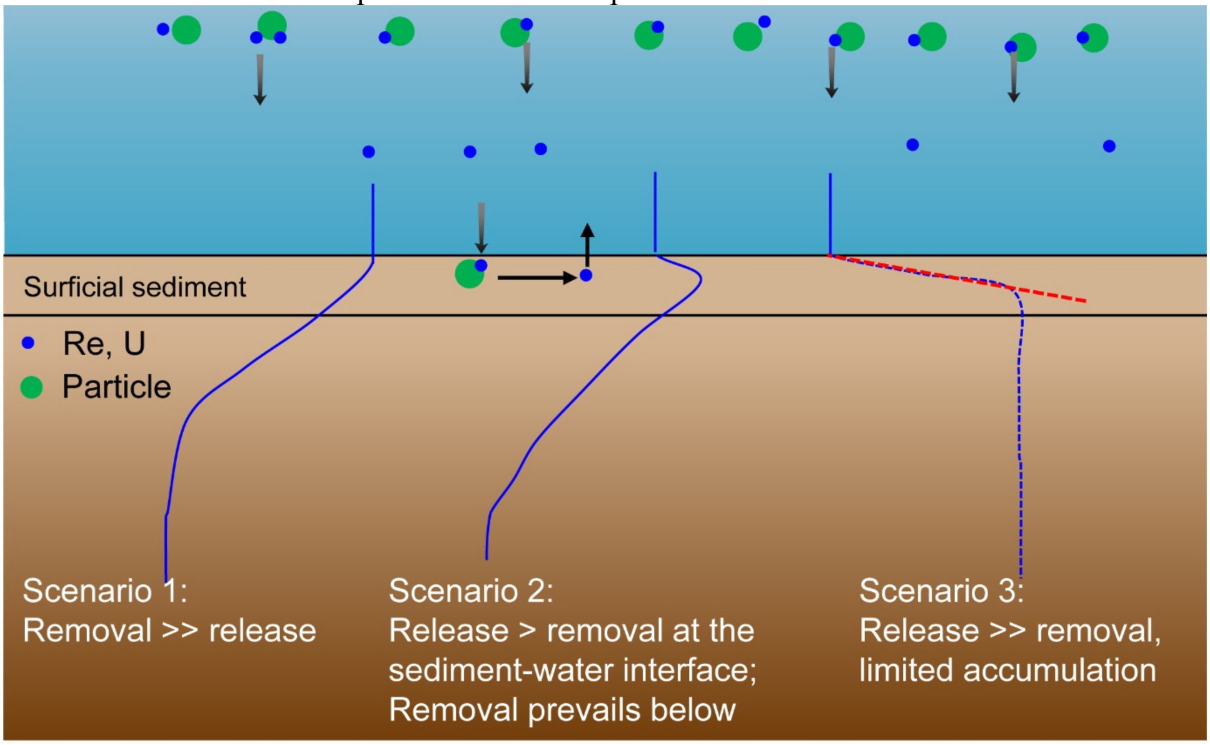

Fig. S6 | Correlations of sedimentary reductive removal of Re (a,c) and U (b,d) versus dissolved oxygen (DO) concentration in the bottom water and penetration depth in the sediment. Triangles and circles denote data collected during summer and winter, respectively.

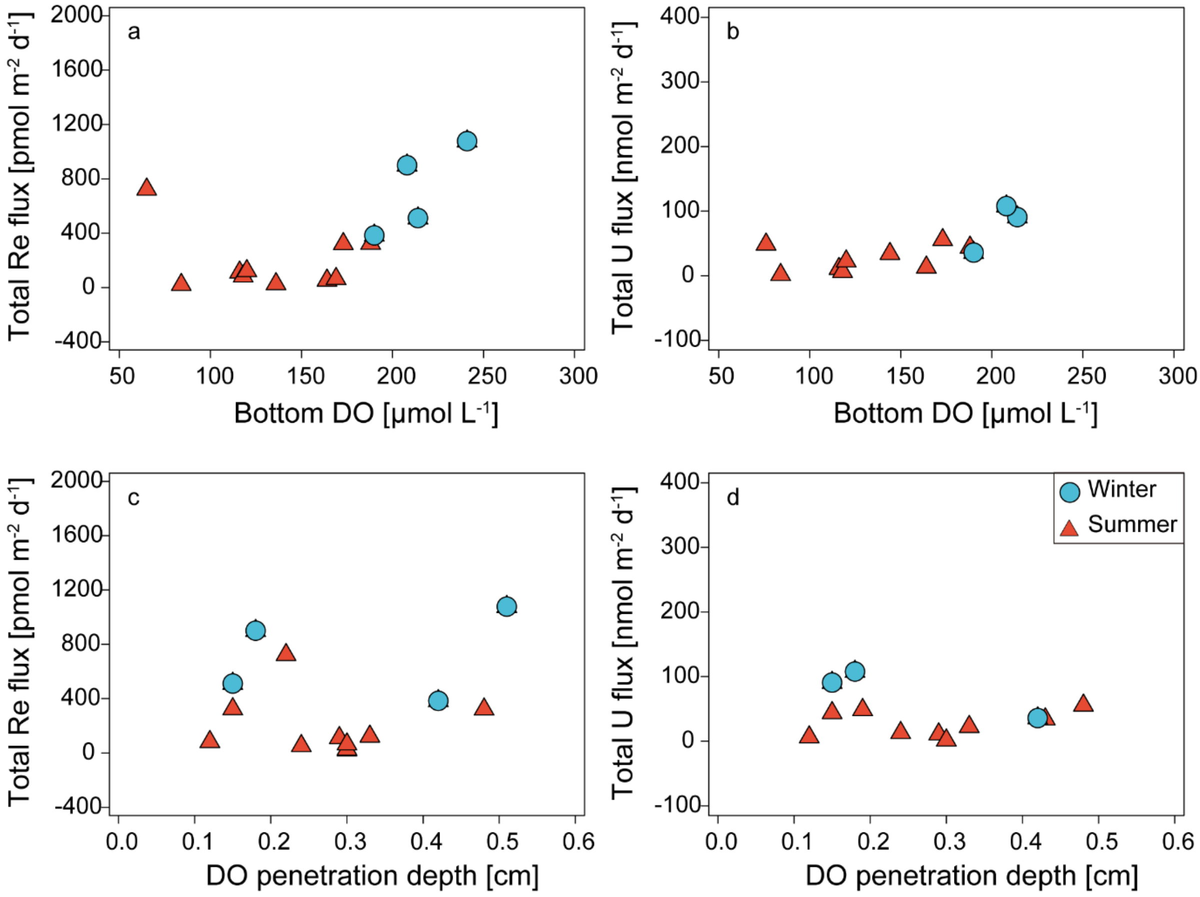

Fig. S7 | Calculation of the concentration gradients of Re (a) and U (b). The concentration gradients were modeled using a linear fit (denoted by red lines) from the surface to the depth where significant gradient change occurs (filled circles). For profiles with distinct zig-zag patterns within the upper few cm, indicating complicated removal-release in the upper sediments, their removal flux was not calculated due to the large uncertainties.

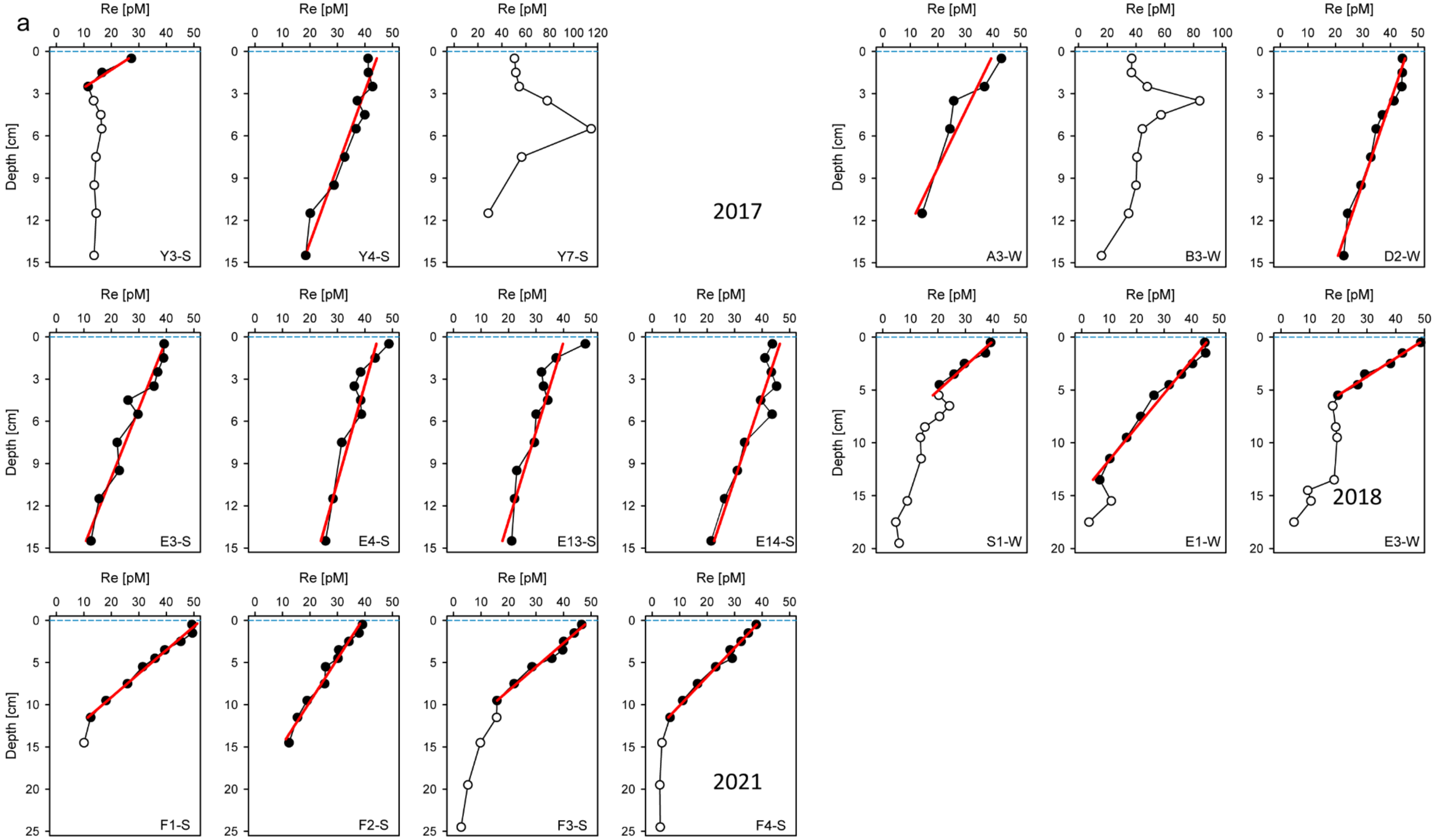

Fig. S7 (continued)

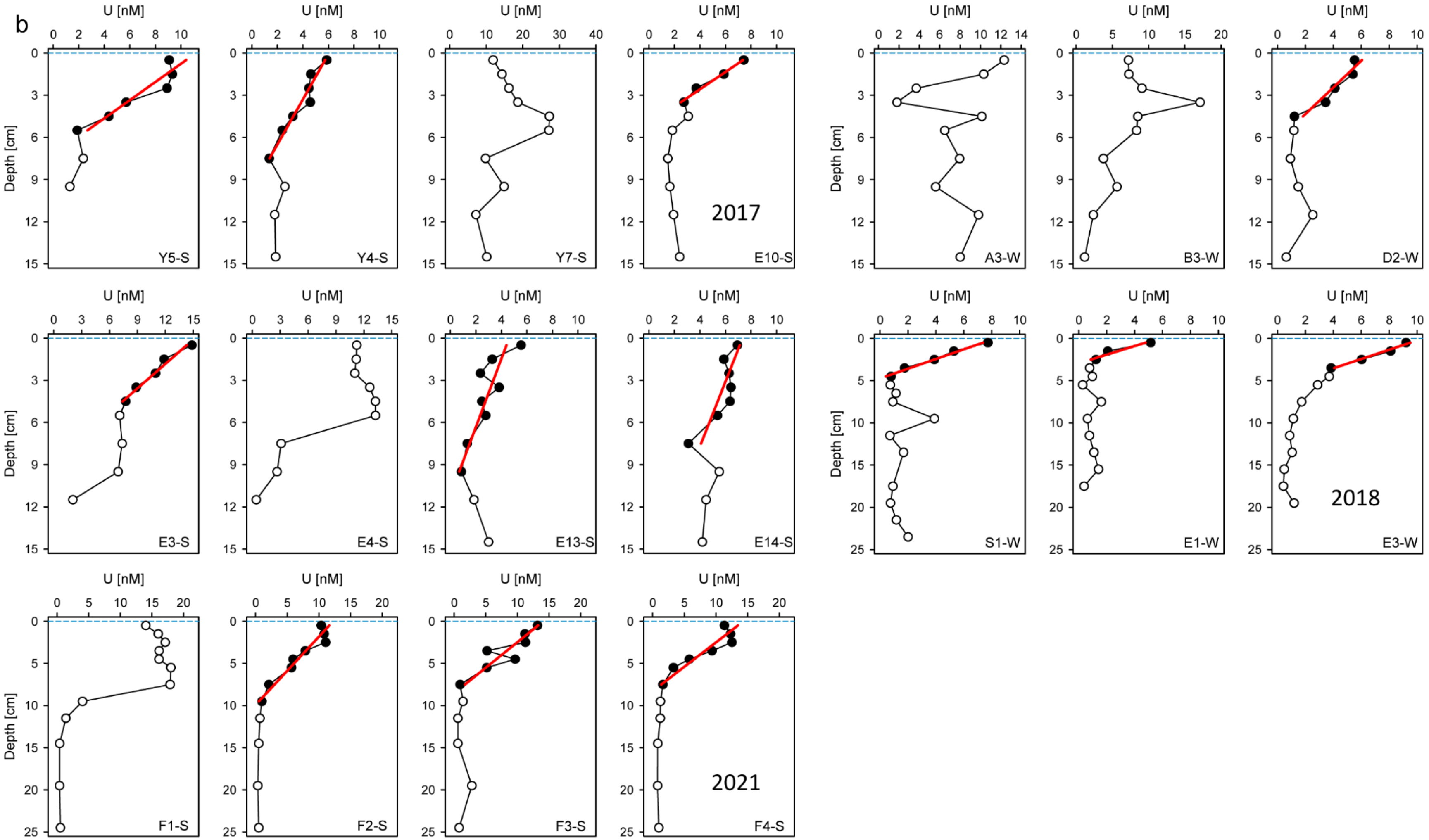

Supplementary References

1. Shi X., *et al.* Large benthic fluxes of dissolved iron in China coastal seas revealed by $^{224}Ra/^{228}Th$ disequilibria. *Geochim. Cosmochim. Acta*, **260:** 49-61 (2019).
2. Wei L., *et al.* Winter mixing accelerates decomposition of sedimentary organic carbon in seasonally hypoxic coastal seas. *Geochim. Cosmochim. Acta*, **317:** 457-471 (2022).
3. Deng K., *et al.* Dominance of benthic fluxes in the oceanic beryllium budget and implications for paleo-denudation records. *Sci. Adv.*, **9:** eadg3702 (2023).
4. Song G., Liu S., Zhu Z., Zhai W., Zhu C. & Zhang J. Sediment oxygen consumption and benthic organic carbon mineralization on the continental shelves of the East China Sea and the Yellow Sea. *Deep Sea Res. II*, **124:** 53-63 (2016).
5. Liu X., Laws E. A., Xie Y., Wang L., Lin L. & Huang B. Uncoupling of Seasonal Variations Between Phytoplankton Chlorophyll a and Production in the East China Sea. *J. Geophys. Res.*, **124:** 2400-2415 (2019).
6. Morford J. L., Martin W. R. & Carney C. M. Rhenium geochemical cycling: Insights from continental margins. *Chem. Geol.*, **324–325:** 73-86 (2012).
7. Morford J. L., Martin W. R., François R. & Carney C. M. A model for uranium, rhenium, and molybdenum diagenesis in marine sediments based on results from coastal locations. *Geochim. Cosmochim. Acta*, **73:** 2938-2960 (2009).
8. Morford J. L., Martin W. R., Kalnejais L. H., François R., Bothner M. & Karle I.-M. Insights on geochemical cycling of U, Re and Mo from seasonal sampling in Boston Harbor, Massachusetts, USA. *Geochim. Cosmochim. Acta*, **71:** 895-917 (2007).
9. Jørgensen B. B., Wenzhöfer F., Egger M. & Glud R. N. Sediment oxygen consumption: Role in the global marine carbon cycle. *Earth-Sci. Rev.*, **228:**

103987 (2022).

10. Kuzyk Z. Z. A., Gobeil C., Goñi M. A. & Macdonald R. W. Early diagenesis and trace element accumulation in North American Arctic margin sediments. *Geochim. Cosmochim. Acta*, **203:** 175-200 (2017).

11. Esch M. E. S., Shull D. H., Devol A. H. & Moran S. B. Regional patterns of bioturbation and iron and manganese reduction in the sediments of the southeastern Bering Sea. *Deep Sea Res. II*, **94:** 80-94 (2013).

12. Grebmeier J. M., Cooper L. W., Feder H. M. & Sirenko B. I. Ecosystem dynamics of the Pacific-influenced Northern Bering and Chukchi Seas in the Amerasian Arctic. *Prog. Oceanogr.*, **71:** 331-361 (2006).

13. Li L*., et al.* Early diagenesis and accumulation of redox-sensitive elements in East Siberian Arctic Shelves. *Mar. Geol.*, **429:** 106309 (2020).

14. Kuzyk Z. Z. A., Macdonald R. W., Stern G. A. & Gobeil C. Inferences about the modern organic carbon cycle from diagenesis of redox-sensitive elements in Hudson Bay. *J. Mar. Syst.*, **88:** 451-462 (2011).

15. Kuzyk Z. Z. A., Macdonald R. W., Johannessen S. C., Gobeil C. & Stern G. A. Towards a sediment and organic carbon budget for Hudson Bay. *Mar. Geol.*, **264:** 190-208 (2009).

16. Eakins B. & Sharman G. Hypsographic curve of Earth's surface from ETOPO1. *NOAA/NGDC*, **5:** 1 (2012).

17. Burdige D. J. Preservation of Organic Matter in Marine Sediments: Controls, Mechanisms, and an Imbalance in Sediment Organic Carbon Budgets? *Chem. Rev.*, **107:** 467-485 (2007).

18. Sheen A. I*., et al.* A model for the oceanic mass balance of rhenium and implications for the extent of Proterozoic ocean anoxia. *Geochim. Cosmochim. Acta*, **227:**

75-95 (2018).

19. Dunk R. M., Mills R. A. & Jenkins W. J. A reevaluation of the oceanic uranium budget for the Holocene. *Chem. Geol.*, **190:** 45-67 (2002).

20. Zhang F*., et al.* Extensive marine anoxia during the terminal Ediacaran Period. *Sci. Adv.*, **4:** eaan8983 (2018).

21. Yang L*., et al.* SLRS-6: River Water Certified Reference Material for Trace Metals and other Constituents. National Research Council of Canada; 2015.

22. Yeghicheyan D*., et al.* A New Interlaboratory Characterisation of Silicon, Rare Earth Elements and Twenty-Two Other Trace Element Concentrations in the Natural River Water Certified Reference Material SLRS-6 (NRC-CNRC). *Geostand. Geoanal. Res.*, **43:** 475-496 (2019).

23. Wang W., Ma L., Evans R. D., Babechuk M. G. & Dang D. H. Quantification of Re and four other trace elements (Ag, Cd, Pd, Zn) in certified reference materials and natural waters. *J. Anal. Atom. Sepctrom.*, **37:** 1471–1483 (2022).

24. Nadeau K*., et al.* NASS-7: Seawater Certified Reference Material for Trace Metals and other Constituents. National Research Council of Canada; 2016.

25. Willie S*., et al.* MESS-4: Marine Sediment Certified Reference Material for total and extractable metal content. National Research Council of Canada; 2014.

26. Schlitzer, R. *Ocean Data View*. https://odv.awi.de, (2020).
